# Frontonasal dysplasia: clinical evaluation on audiological and brainstem electrophysiological profiles

**DOI:** 10.1590/S1808-86942011000500013

**Published:** 2015-10-22

**Authors:** Melissa Zattoni Antoneli, Célia Maria Giacheti, Kátia Flores Genaro, Neivo Luiz Zorzetto, Antonio Richieri-Costa

**Affiliations:** 1 Speech therapist. Master's degree; author; 2 Speech therapist. Associate professor; 3 Speech therapist. Associate professor; 4 Anatomist. Associate and Full professor; 5 Physician. Associate professor. Geneticist

**Keywords:** evoked potentials, auditory, brain stem, hearing, hypertelorism

## Abstract

**Abstract:**

Frontonasal dysplasia (FND) is a rare malformative complex affecting the frontal portion of the face, the eyes and the nose; it may occur singly or associated with other clinical signs. No systematic studies describing hearing in this condition were found.

**Aim:**

To evaluate hearing sensitivity and sound stimulus conduction from cochlea to brainstem in patients with clinical signs of FND.

**Methods:**

21 patients with isolated or syndromic FND were submitted to a clinical (otological/ vestibular antecedents and otoscopy) and instrumental (pure tone and speech audiometry, tympanometry and brainstem auditory evoked response) hearing evaluation.

**Design:**

A clinical, cross-sectional observational prospective study.

**Results:**

Hearing thresholds were normal in 15 (70%) patients, abnormal in 5 (25%), mostly with conductive hearing loss; one patient did not cooperate with testing. The tympanometric curve was type A in 30 (72%) ears, type C in 5 (12%), type As in 4 (9%) and type B in 3 (7%). The auditory brainstem response (ABR) showed no abnormalities.

**Conclusion:**

Patients with FND showed no abnormalities in the auditory system from cochlea to brainstem in this study. Mild conductive hearing loss found in some is probably related to cleft palate. Further evaluation of hearing pathways at higher levels is recommended.

## INTRODUCTION

Frontonasal dysplasia (FND) is a rare malformation characterized mainly by abnormalities in the eyes, nose, and frontal portion of the face. Clinical manifestations vary, and may include ocular hypertelorism, a wide nasal base and flat broad nose, a missing tip of the nose, a vertical cleft along the middle of the face affecting the nose and/or upper lip, a low-set frontal hairline (also known as “widow's peak” frontal hairline), and an bifid anterior cranium occultum^1^. This condition is also referred to as clefts 0-14 in Tessier's classification^2^.

Researchers have reported several clinical findings in FND; involvement of the face may range from mild hypertelorism to a middle facial cleft^1^,^3^,^4^, and it may occur singly, associated with other malformations, or as part of syndromes including or not central nervous system (CNS) anomalies.

We found only case reports of FND with hearing loss in the Brazilian and international literature. Specific studies on hearing in FND were not present in the papers we compiled.

Sensorineural hearing loss was reported in a female child aged 11 months diagnosed with FND and renal agenesis^5^.

Four FND patients were studied, of which one had conductive hearing loss. This patient also presented cleft lip and palate, agenesis of the corpus callosum, delayed neuromotor development, and mental retardation^6^.

A study has been published on a mother and daughter with craniofacial syndrome and limited abduction of the hips and shoulders^7^. The mother had axillary pterygium, cleft lip and palate, congenital stapes fixation in the left ear, and right ear sensorineural hearing loss.

Conductive hearing loss was reported in a 4-year-old girl with FND and no CNS anomalies. Otoscopy revealed otitis media with effusion, and immittance testing showed a tympanometric curve without a compliance peak and bilateral absence of the stapedial reflex^8^.

Another case was a patient with FND, neuronal migration error, upper and lower limb lymphedema, and mild delayed neuropsychomotor development^9^. Audiometry revealed mild low frequency and moderate high frequency hearing loss; although the type of loss was not reported, the sloping audiometric curve suggested sensorineural hearing loss.

If the phenotype of a given condition is known, anomalies that are not obvious but that are part of the condition should be investigated. The focus of most papers on FND has been the physical features of this disease; however, functions such as speech and hearing have not been explored in depth.

Because of the importance of hearing for communications, and taking into account that individuals with craniofacial malformations are at risk for hearing, speech, and language disorders, it is relevant to check the function of the auditory system to diagnose possible conditions and reflect on treatment in these cases.

Thus, the purpose of this study was to investigate auditory function in subjects with clinical signs of single or syndromic FND, and to measure the acuity of hearing and the integrity of pathways that conduct sound stimuli to the brainstem.

## MATERIAL AND METHODS

The study was started following approval by the institutional review board; it met the requirements of Resolutions 196/96 and 251/97, and the approval protocol numbers were 368/2006 (4 December 2006) and 140/2010 (8 July 2010). All study subjects or their representatives signed a free informed consent form.

The study sample comprised 21 subjects with a diagnosis of FND. The age of patients ranged from 7 to 42 years; the mean age was 19 years, the median was 16 years, and the mode was 8 years. There were 14 female subjects (66%) and seven male subjects (34%), totaling 21 subjects. Fourteen subjects (66%) were diagnosed with single FND and seven (34%) with syndromic FND; two of these (9.6%) were diagnosed with the craniofrontonasal syndrome, one (4.8%) with the cerebrofrontofacial syndrome, one (4.8%) with the oculoauriculofrontonasal syndrome, one (4.8%) with alar cleft and FND, one (4.8%) with agenesis of the corpus callosum, basal encephalocele, ocular anomalies and FND, and one (4.8%) with agenesis of the corpus callosum, encephalocele, mental retardation and FND. In 21 study subjects, seven (34%) had structural anomalies of the corpus callosum - four (19.2%) with agenesis, one (4.8%) with hypoplasia, one (4.8%) with a lipoma, and one (4.8%) with a pericallosal lipoma. Nine subjects (43%) had cleft palate that had already been treated surgically at the time of the evaluation, and 12 (57%) had normal palates.

The audiologic evaluation consisted of five procedures: the clinical history, an ontological inspection (to see if there were any impediments to other tests, such as excess wax or foreign bodies), immittance testing, pure tone audiometry, and brainstem auditory evoked potentials (BAEP). A Hortmann Neuro-otometrie BERAmodul device was used for recording BAEP. The following parameters were used: 0.1 millisecond (ms) clicks, and 24 clicks presented per second. The analysis time was 10 ms, waves were recorded with the sum of 1,000 stimuli with alternated polarity; the study intensities were 80 dB when peripheral hearing was normal and the tracing was clearly visible; and were 90, 100 and 110 dB in cases of peripheral hearing loss or when tracings were altered at the initial 80 dB intensity; three recordings were made for each intensity. Stimuli were presented using Hortmann Neuro-Otometrie Beyerdynamic DT48 over-the-ear phones. During the test, patients were lying on a comfortable semi-reclined bed in an acoustic room.

The electrophysiologic threshold was not measured, as the purpose of this study was to establish the integrity of auditory pathways, and not auditory sensitivity. BAEP tests were analyzed and compared with a normal reference. Normal parameters for analyzing latencies were the values obtained by biological calibration of the equipment.

## RESULTS

The audiometric results were normal in 15 subjects (70%) of 21; the audiometric results were abnormal in five subjects (25%), and one subject (5%) did not collaborate with testing. The findings were mild bilateral conduction hearing loss in two subjects (10%), mild unilateral conduction hearing loss in one subject (5%), mild unilateral isolated hearing loss at 6 and 8 kHz in one subject (5%), and mild unilateral isolated hearing loss at 8 kHz in one subject (5%).

Forty ears were tested, of which 33 (82.5%) had normal audiometric thresholds and seven (17.5%) had altered audiometric thresholds.

Table 1 shows the mean audiometric thresholds per frequency.

**Table 1:** Distribution of minimum, maximum, and mean audiometric threshold values at each frequency for 40 tested ears.

Frequencies		Values (dB)	
(Hz)	Minimum	Maximum	Mean
0,25	5	30	09,625
0,5	5	35	12,625
1	0	20	07,875
2	0	25	08,875
3	0	20	08,750
4	0	40	11,125
6	0	30	12,875
8	0	40	11,625

The results of tympanometry in 42 ears of 21 tested subjects were a type A curve in 30 ears (72%), a type C curve in five ears (12%), a type Ar curve in four ears (9%), and a type B curve in three ears (7%). Acoustic reflexes were present in 24 ears (57%), absent in 12 ears (29%), non-systematic in three ears (7%), and not measured in three ears (7%).

BAEP findings were similar among the study sample; no altered neural conduction of auditory stimuli up to the brainstem was observed. The wave I, II, and III absolute latency values and the I-III, III-V, and I-V interpeak values were within normal limits.

Table 2 presents the descriptive statistics of the BAEP parameters.

**Table 2:** Distribution of minimum, maximum, and mean values, and the standard deviation, for brainstem auditory evoked potentials per ear.

Side	Variables	Values (ms)				
		N	Minimum	Maximum	Mean	SD
	I	21	1,8	2,0	1,92	0,070
	III	21	3,8	4,1	3,97	0,095
Right	V	21	5,7	6,1	5,88	0,124
Ear	I-V	21	3,8	4,2	3,96	0,096
	III-V	21	1,8	2,0	1,91	0,067
	I-III	21	1,9	2,2	2,05	0,067
	I	21	1,8	2,0	1,91	0,275
	III	21	3,8	4,1	3,96	0,510
Left	V	21	5,7	6,1	5,88	0,525
Ear	I-V	21	3,8	4,2	3,97	0,505
	III-V	21	1,8	2,1	1,92	0,141
	I-III	21	1,9	2,2	2,03	0,604
	V - Inter-ear difference	21	0	0,2	0,04	0,067

## DISCUSSION

Previous studies have reported in detail the phenotype, and the variability of craniofacial and CNS malformations in FND^1^,^3^,^4^,^10^, ^11^, ^12^, ^13^, ^14^; few of these studies, however, have described the functionality of these structures.

Hearing has not been investigated in depth, and therefore there are no systematic data to compare with the present study. We found a 2:1 female to male ratio in our study sample, which differs from previously published data^11^. Although this ratio is significant given the low incidence of FND in the population^15^,^16^, this number is not sufficient to characterize its demography and discuss the sex ratio.

There is a known relationship between cleft palate and hearing loss - mostly conduction, but occasionally sensorineural hearing loss^17^,^18^. Furthermore, cleft palate may occur in FND patiets^19^, ^20^, ^21^, ^22^, although the frequency of this finding has not been reported in the literature.

FND with cleft palate was present in about half of our study sample (43%); this finding is probably a peculiarity of our series, as our sample was taken from a center that specializes in the treatment of cleft lip and palate. All of these patients had already been treated surgically, and therefore had good velopharyngeal function.

There are only five FND patients with hearing loss described in the literature^5^, ^6^, ^7^, ^8^, ^9^, of which three (60%) had sensorineural hearing loss and two (40%) had conduction hearing loss. Only one of these five patients with hearing loss had single FND; the other four had syndromic FND or FND associated with other anomalies. One of the two subjects with conduction hearing loss had agenesis of the corpus callosum and cleft palate, while the other had otitis media with effusion, which were possible etiological factors. The three subjects with sensorineural hearing loss had other anomalies: one with renal agenesis; one with the craniofrontonasal syndrome, mental retardation, delayed neuropsychomotor development, cleft palate, and joint hypomobility; and one with neuron migration error, limb lymphedema, and mild delayed cognitive and speech development. Anomalies of the corpus callosum have not been described in any of these reported cases with sensorineural hearing loss.

The presence of anomalies in the skull, face, and CNS, as well as possible malformations affecting peripheral and central auditory pathways and/or other associated anomalies, which have been reported in most of these published cases, raise questions about possible etiologies for hearing loss, as only one of the FND cases in the literature had single FND without CNS anomalies. On the other hand, studies describing auditory anomalies in FND cases have been isolated case reports, which makes it difficult to infer whether the presence, type, and degree of hearing loss is part of a pattern or a non-systematic occurrence.

In the present study of 21 FND cases, most of the subjects had normal hearing (76%); five subjects (24%) had hearing loss as demonstrated with audiometry, of which three (60%) had conduction hearing loss, and (40%) had sensorineural hearing loss. Of these five cases with hearing loss, two had single FND (40%) and three had syndromic FND (60%).

A comparison showed that cleft palate was present in about 60% of hearing loss cases and in 30% of normal hearing subjects. A corpus callosum anomaly was found in 40% of patients with hearing loss, and in 30% of normal hearing subjects. These two anomalies were more frequent in patients with hearing loss - in particular cleft palate. Although hearing loss related with recurring otitis has been reported as being more frequent in patients with agenesis of the corpus callosum compared to normal individuals^23^, in the present study this type of alteration appeared to be related with cleft palate, which is a known risk factor for conduction hearing loss^17^,^24^.

Few of the study subjects had altered peripheral auditory pathways (the middle ear and cochlea). Neuronal conduction in the auditory pathways assessed by BAEP appeared to be intact from the distal portion of the 8^th^ cranial nerve to the superior olivary complex in the brainstem. Although conduction disorders classically increase absolute latencies in BAEP waves, this finding was not seen in our sample. It is possible that since these findings were mild in all patients, they may not have been sufficiently consistent to cause detectable delayed sound conduction by BAEP, of that this delay was not clinically meaningful since latency values were close to the maximum normal limit.

Our series may be considered heterogeneous in terms of the genetic diagnoses, the clinical features, and age. However, the audiologic findings - which were similar among subjects - did not seem to reflect this heterogeneity.

The present study underlines the possibility and importance of evaluating higher levels of the auditory system - including structures such as the cerebral cortex - with other electrophysiologic and behavioral tests, such as middle and long latency potentials and auditory processing tests. The normal results of the auditory system in our study may be considered as a starting point for this purpose. Molecular studies may be used in future studies to investigate genetic mutations, and may add much information because of recent advances in understanding the etiology of FND^22^,^25^,^26^.

## CONCLUSION

The basic audiologic evaluation - audiometry and immittance testing - revealed that auditory acuity was decreased in 33% of the study sample, consisting mostly of mild bilateral hearing loss. There were no auditory pathway disorders associated with sound stimulus conduction to the brainstem, as evaluated in the BAEP.

FND and associated factors, such as CNS alterations, initially had no relation with hearing loss. The only factor that appears to be related with hearing loss is cleft palate, which may occur in isolation or associated with FND or other genetic conditions. It is a well-known etiological factor for conductive hearing loss.

We concluded that patients with single or syndromic FND had no auditory alterations as part of their clinical manifestations, although these individuals are at risk of hearing loss. Further studies with more FND subjects and audiologic procedures are needed so that hearing profiles may be defined.
